# Modeling Fibrin Accumulation on Flow‐Diverting Devices for Intracranial Aneurysms

**DOI:** 10.1002/cnm.3883

**Published:** 2024-11-05

**Authors:** Juan R. Cebral, Fernando Mut, Rainald Löhner, Laurel Marsh, Alireza Chitsaz, Cem Bilgin, Esref Bayraktar, David Kallmes, Ramanathan Kadirvel

**Affiliations:** ^1^ Bioengineering Department George Mason University Fairfax Virginia USA; ^2^ Physics Department George Mason University Fairfax Virginia USA; ^3^ Department of Radiology Mayo Clinic Rochester Minnesota USA; ^4^ Department of Neurosurgery Mayo Clinic Rochester Minnesota USA

**Keywords:** cerebral aneurysms, coupling, fibrin accumulation, flow diversion, thrombosis

## Abstract

The mechanisms leading to aneurysm occlusion after treatment with flow‐diverting devices are not fully understood. Flow modification induces thrombus formation within the aneurysm cavity, but fibrin can simultaneously accumulate and cover the device scaffold, leading to further flow modification. However, the interplay and relative importance of these processes are not clearly understood. A computational model of fibrin accumulation and flow modification after flow diversion treatment of cerebral aneurysms has been developed under the guidance of in vitro experiments and observations. The model is based on the loose coupling of flow and transport‐reaction equations that are solved separately by independent codes. Interaction or reactive terms account for thrombin production from prothrombin stimulated by thrombogenic metallic wires and inhibition by antithrombin as well as fibrin production from fibrinogen stimulated by thrombin and flow shear stress, and fibrin adhesion to device wires and already attached fibrin. The computational model was demonstrated and tested on idealized vessel and aneurysm geometries. The model was able to reproduce the salient features of fibrin accumulation after the deployment of flow‐diverting devices in idealized in vitro models of cerebral aneurysms. Namely, fibrin production in regions of high shear stress, initial accumulation at the inflow zone, and progressive occlusion of the device and corresponding flow attenuation. The computational model linking flow dynamics to fibrin production, transport, and adhesion can be used to investigate and better understand the effects that lead to fibrin accumulation and the resulting aneurysm inflow reduction and intra‐aneurysmal flow modulation.

## Graphical abstract

A computational model of fibrin accumulation and associated flow modification after flow diversion treatment of cerebral aneurysms has been developed under the guidance of in vitro experiments and observations. The model was able to reproduce the salient features of fibrin accumulation patterns observed after deployment of flow‐diverting devices in idealized in vitro models of cerebral aneurysms.

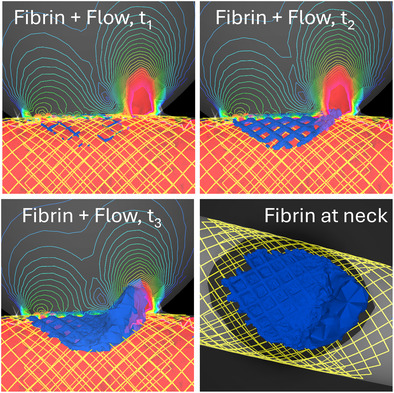

## 1 Introduction

Wide‐necked cerebral aneurysms present a significant challenge in neurovascular treatment due to their complex morphology and the limitations of traditional therapeutic techniques such as coiling and clipping [1]. Flow‐diverting (FD) devices have emerged as an innovative solution, offering a minimally invasive approach to treating these challenging aneurysms and have become a common treatment option for many intracranial aneurysms [2, 3]. These devices are designed to be placed across the neck of the aneurysm, redirecting blood flow away from the aneurysm sac and encouraging progressive thrombosis within the aneurysm [4]. However, nearly one‐fifth of aneurysms do not close after FD placement [5]. These aneurysms may remain open or incompletely occluded for a long time, requiring close monitoring and possibly additional interventions [6, 7, 8].

The flow conditions created by FD devices, namely reduced inflow jet, lower wall shear stress, flow redirection away from the sac, disruption of vortices, and flow stagnation are all designed to promote the occlusion of the aneurysm and the integration of the device into the vessel wall. Hence, the outcome of the FD procedure depends largely on the posttreatment flow environment that promotes thrombus formation and aneurysm occlusion. Although the prevalent flow conditions created by FD devices are valuable in assessing the likelihood of a successful procedure [9], predictive models based on only hemodynamic parameters are limited and their predictive accuracy peaks at less than 80% [10, 11, 12, 13].

Aneurysm occlusion after the deployment of flow diverters is a complex process that results from the interplay of several factors [4]. Initially, the flow diverter disrupts the blood flow entering the aneurysm, significantly reducing the inflow jet and shear stress on the aneurysm wall. This flow disruption leads to the formation of a thrombus within the aneurysm cavity, as blood stasis occurs. Concurrently, fibrin, a protein involved in blood clotting, accumulates across the cells of the flow diverter that covers the aneurysm orifice [14]. This fibrin scaffold is crucial for stabilizing the initial clot. Over time, endothelial cells proliferate and migrate across the surface of the flow diverter, a process known as endothelialization. This endothelial layer further seals the aneurysm from the parent artery, integrating the device into the vessel wall and providing a permanent barrier to blood flow [15]. Each of these processes occurs on a different timescale and it is not entirely clear which process takes precedence in a given aneurysm [16, 17].

Developing computational models that incorporate these mechanisms could not only contribute to our detailed understanding of the occlusion and healing process after treatment but also to improve outcome assessment and optimization of interventional procedures as well as improve the design of FD devices. Mathematical models, consisting of ordinary differential equations (ODEs), have been used to evaluate the coagulability of blood samples by describing the concentrations of various clotting factors over time [18]. These models are useful for comparing results with major coagulation assays such as the Thrombin Generation Assay (TGA) and Endogenous Thrombin Potential (ETP). However, they do not quantify thrombus growth mechanisms. To address this problem, more complex numerical models include advection and diffusion terms for each coagulation reaction equation to simulate blood flow and platelet aggregation dynamics [19], and use a continuous approach such as the Navier–Stokes equations [20, 21, 22], or discrete methods such as Dissipative Particle Dynamics [23], or a hybrid strategy where blood is modeled as a continuum and platelets as flowing particles [24]. Continuous models usually consider the effect of the clot on the blood flow through either a porosity force [20, 22] or a modified viscosity [21] and hybrid methods through a particle drag force [24]. Although these complex models provide a quantitative understanding of thrombus growth within the aneurysm cavity, they have mainly focused on platelet aggregation and have not studied fibrin accumulation in detail [22, 24, 25]. Recent studies have considered thrombin‐fibrin interactions in experimental in vitro models but have not analyzed fibrin accumulation on device scaffolds [26]. As such, our work focuses on the development of a computational model of fibrin accumulation and thrombus formation after FD implantation (guided by corresponding experimental models) that can be used to analyze the processes leading to complete or incomplete aneurysm occlusions.

## 2 Methods

### 2.1 Conceptual Model

Thrombus formation and clotting are driven by the coagulation cascade which is a complex network of interactions between approximately 80 elements or chemical species dissolved in the blood plasma as well as formed elements, most importantly platelets and erythrocytes [27]. Clots can be thought of as mainly composed of a network of activated platelets and fibrin fibers that adhere to one another forming a net that traps erythrocytes. Although platelets and erythrocytes play an important role in thrombus formation and flow alteration within the aneurysm cavity, fibrin alone can also accumulate at FD wires and disrupt the inflow into the aneurysm. As such, this study focuses on the process of fibrin accumulation in the absence of platelets and erythrocytes.

**FIGURE 1:**
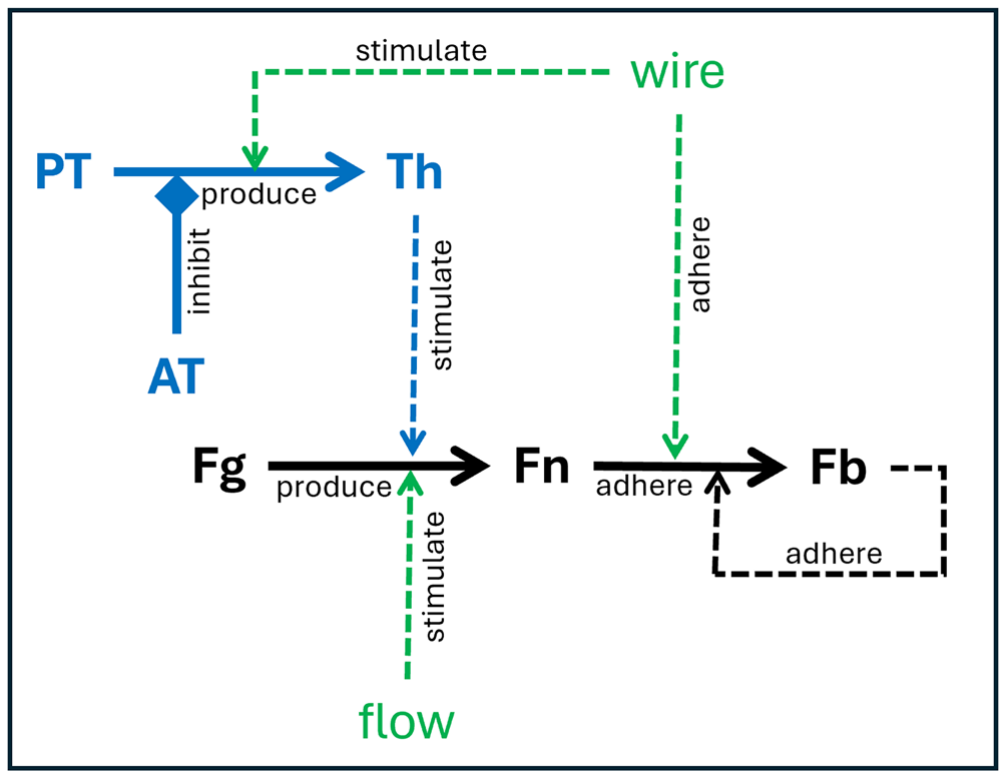
Simplified fibrin production and accumulation model. PT = prothrombin, AT = antithrombin, Th = thrombin, Fg = fibrinogen, Fn = fibrin (free), Fb = bounded fibrin.

Fibrin (Fn) is a formed element that consists of polymeric fibers that can adhere to thrombogenic elements such as metallic device wires, or collagen fibers exposed by vessel injury or de‐endothelialization, or to other attached or bounded fibrin (Fb) fibers [28]. Fibrin is produced by the cleavage of fibrinogen (Fg) proteins that are dissolved in the blood plasma. This is stimulated by the presence of thrombin (Th) [29] and by flow shear stress as will be shown later. In turn, thrombin is produced from prothrombin (PT) in the presence of a thrombogenic foreign element (e.g., metallic wires, exposed collagen) and inhibited by anti‐thrombin (AT). This simplified network of interactions is schematically represented in Figure 1 and is the basis of the conceptual model of this study.

### 2.2 Mathematical Model

#### 2.2.1 Fluid Flow

The mathematical model is based on a continuum approximation [20, 30]. The flow of plasma is described by the incompressible Navier–Stokes equations:

(1) ∇∙u=0

(2) $$\rho \left(\frac{\partial u}{\partial t}+u∙\nabla u\right)=-\nabla p+\nabla ∙\tau +F$$

where ρ is the fluid (plasma) density, p is the pressure, u is the velocity, and F is represents external forces. The deviatoric shear stress tensor τ for a Newtonian fluid is given by:

(3) $$\tau_{\mathrm{ij}}=2\mu \;e_{\mathrm{ij}}$$

where μ is the viscosity and the strain rate tensor is:

(4) $$e_{\mathrm{ij}}=\frac{1}{2}\left(\frac{\partial u_{i}}{\partial x_{j}}+\frac{\partial u_{j}}{\partial x_{i}}\right)$$

The magnitude of shear stress at a point in the fluid can be calculated as:

(5) $$\tau =\mu \;\dot{\gamma }$$

where $\dot{\gamma }$ is the second invariant of the strain rate tensor:

(6) $$\dot{\gamma }=2\sqrt{e_{\mathrm{ij}}e_{\mathrm{ij}}}$$

The flow alteration caused by the accumulation of fibrin at the device wires is modeled as a porous force that depends on the concentration of bounded fibrin attached to the wires (Darcy's term):

(7) $$F=-\pi \;\phi \left(C_{F_{b}}/C_{F_{b0}}\right)\;u$$

where π is a porosity parameter (inverse of the permeability), $C_{F_{b}}$ is the concentration of bounded or attached fibrin, $C_{F_{b0}}$ is a threshold below which the force goes to zero, and ϕ is a smooth switching or step (Hill) function that represents the inverse of the permeability of the accumulated fibrin:

(8) $$\phi \left(x\right)=\frac{x^{n}}{1+x^{n}}$$

and the parameter n controls the steepness of the step function.

#### 2.2.2 Species Transport and Interactions

The conceptual model described in Figure 1 is mathematically represented by transport (convection and diffusion) and reaction equations for six species: prothrombin, antithrombin, thrombin, fibrinogen, (free) fibrin, and bounded fibrin. These equations can be written as:

(9) $$\frac{\partial C_{i}}{\partial t}+u∙\nabla C_{i}=D_{i}\;\nabla^{2}C_{i}+S_{i}$$

where $i=\mathrm{PT},\mathrm{AT},\mathrm{Th},F_{g},F_{n},F_{b}$ denotes the species, $C_{i}$ is the concentration of species i and $D_{i}$ is the diffusivity. The source term ($S_{i}$) assumes different forms for the different species and accounts for the different interactions and processes represented in Figure 1:

(10) $$S_{\mathrm{PT}}=-K_{\mathrm{wt}}\;\phi \left(r/r_{0}\right)\;C_{\mathrm{PT}}$$

(11) $$S_{\mathrm{AT}}=-K_{\mathrm{at}}C_{\mathrm{AT}}C_{\mathrm{Th}}$$

(12) $$S_{\mathrm{Th}}=K_{\mathrm{wt}}\;\phi \left(r/r_{0}\right)\;C_{\mathrm{PT}}-K_{\mathrm{at}}C_{\mathrm{AT}}C_{\mathrm{Th}}$$

(13) $$S_{F_{g}}=-K_{\mathrm{th}}\frac{C_{T_{h}}}{K_{m}+C_{F_{g}}}\;C_{F_{g}}-K_{\mathrm{ss}}\phi \left(\tau /\tau_{0}\right)C_{F_{g}}$$

(14) $$S_{F_{n}}=K_{\mathrm{th}}\frac{C_{T_{h}}}{K_{m}+C_{F_{g}}}\;C_{F_{g}}+K_{\mathrm{ss}}\phi \left(\tau /\tau_{0}\right)C_{F_{g}}-K_{\mathrm{wa}}\phi \left(r/r_{0}\right)C_{F_{n}}-K_{b}C_{F_{b}}C_{F_{n}}$$

(15) $$S_{F_{b}}=K_{\mathrm{wa}}\phi \left(r/r_{0}\right)C_{F_{n}}+K_{b}C_{F_{b}}C_{F_{n}}$$

where $K_{\mathrm{wt}},K_{\mathrm{at}},K_{\mathrm{th}},K_{\mathrm{ss}},K_{\mathrm{wa}},$ and $K_{b}$ are rate constants, ϕ is the Hill step function of Equation (8), and $K_{m},\tau_{0}$, and $r_{0}$ are thrombin, shear stress, and wire distance threshold values, respectively. The first term in Equation (12)—source term for thrombin—represents the production of thrombin from prothrombin stimulated by nearby metallic wires and the second term is the inhibition of this production by antithrombin. The first term in Equation (14)—source term for fibrin—accounts for the production of fibrin from fibrinogen stimulated by thrombin, the second term is the production of fibrin from fibrinogen when the flow shear stress is above a certain threshold, the third term represents the adhesion of free fibrin to nearby device wires (i.e., conversion of free fibrin into bounded fibrin when the distance to the wire is below a threshold), and the last term is the adhesion of free fibrin to other bounded fibrin.

### 2.3 Numerical Model

#### 2.3.1 Solution Strategy

In the mathematical model described above, there is a two‐way coupling between the fluid and species equations. The fluid equations depend on the bounded fibrin concentration through the porosity force term, and the species equations depend on the fluid velocity through the convective terms and the fibrin production source term that depends on the fluid shear stress. However, the characteristic response times of the two systems (fluid flow and species transport and production) are quite different. The fluid responds very fast to changes in the applied loads or geometries (in a fraction of a second), while the species response (accumulation) is much slower (in the order of minutes). This suggests a solution strategy for the coupled problem based on a loose coupling approach where the two problems are solved separately by independent codes that exchange information.

#### 2.3.2 Computational Mesh

**FIGURE 2:**
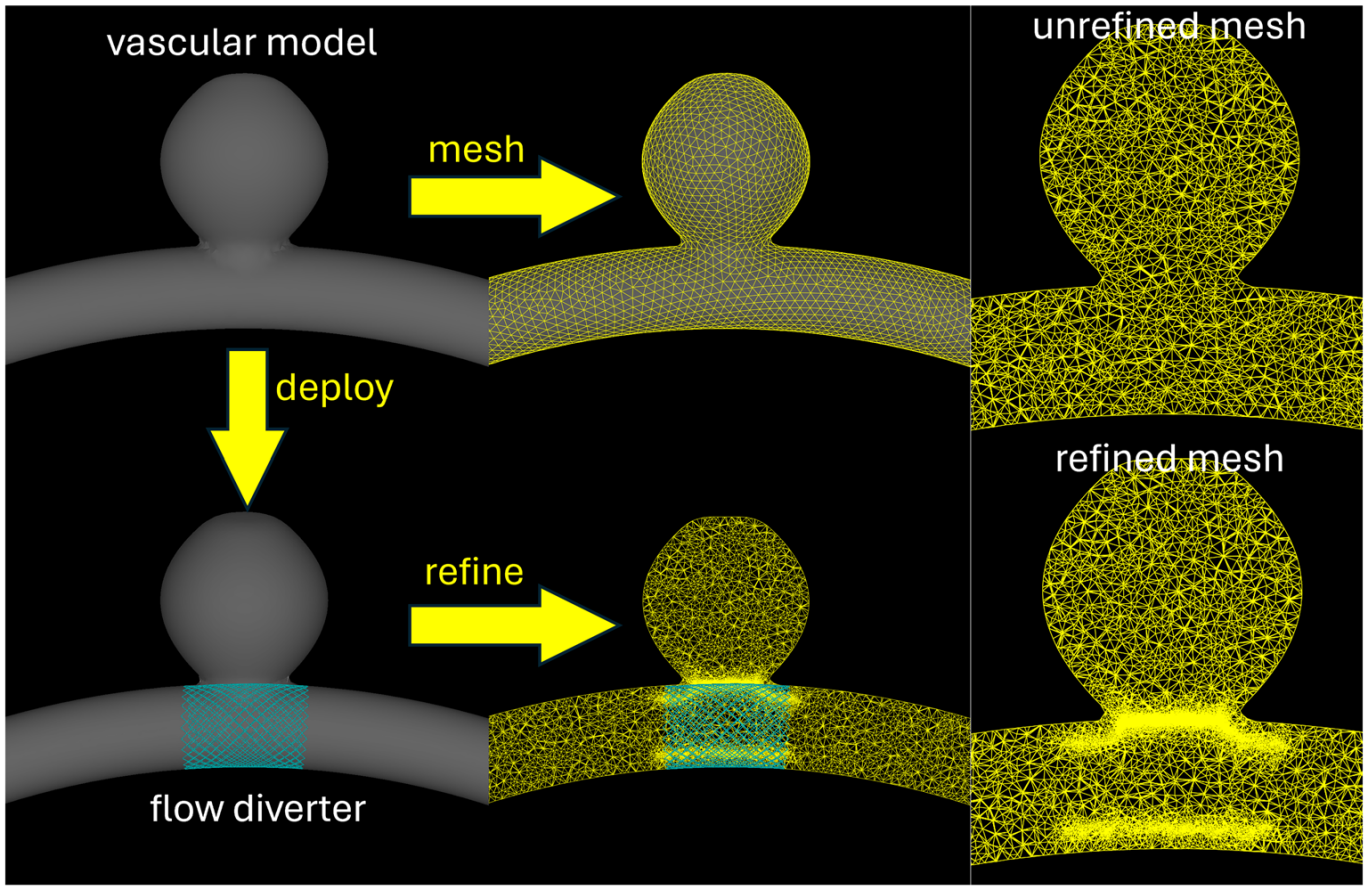
Computational mesh adaptively refined around deployed FD device wires.

To model complex vascular models space discretization is based on unstructured grids composed of tetrahedral elements, while FD devices are modeled with an immersed boundary approach previously described [31]. Briefly, given a water‐tight surface triangulation of the vascular model, a volumetric tetrahedral grid is generated using an advancing front method. Next, the FD device is deployed within the vascular model by expanding a cylinder along the vessel skeleton until it contacts the wall, and subsequently the device wire design is mapped to the cylindrical surface, considering possible foreshortening effects [32]. The device wires are represented by a series of overlapping spheres that are used to identify edges of the mesh elements which are cut by the device where wall boundary conditions will be imposed. Since the wires are very thin compared to the vascular dimensions, the mesh is then adaptively refined around the wires (i.e., elements cut by the device wires) to properly resolve the wires [33]. The process is illustrated in Figure 2.

#### 2.3.3 Flow Solver

The fluid flow equations are integrated in time using a fractional step approach. Briefly, the velocity and pressure are advanced in three steps:

- *Advective‐diffusive velocity prediction* $u^{n}\to u^{*}$:

Discretizing the momentum Equation (2) in time, introducing an intermediate predicted velocity $u^{*}$, and evaluating the pressure, porous, and viscous terms implicitly:

(16) $$\rho \left(\frac{u^{n+1}-u^{*}+u^{*}-u^{n}}{\Delta t}\right)+\rho \;u^{n}∙\nabla u^{n}=-\nabla p^{n+1}+\nabla \mu \nabla u^{*}-\pi \phi u^{*}$$

which can be split into two parts:

(17) $$\rho \left(\frac{u^{n+1}-u^{*}}{\Delta t}\right)=-\nabla \left(p^{n+1}-p^{n}\right)$$

and

(18) $$\left[\frac{\rho }{\Delta t}-\nabla \mu \nabla +\pi \phi \right]\;\left(u^{*}-u^{n}\right)=-\rho \;u^{n}∙\nabla u^{n}-\nabla p^{n}+\nabla \mu \nabla u^{n}-\pi \phi u^{n}$$

where the implicit terms have been moved to the left‐hand side and the explicit terms to the right. The predicted velocity is found with iterative solvers that converge quickly, particularly when the porosity dominates. Furthermore, the stability condition in this scheme is dictated only by the advective terms that are integrated explicitly:

(19) Δt<h/∣u∣

where h represents the element size.

b *Pressure correction* $p^{n}\to p^{n+1}$:

Assuming a divergence‐free velocity field at step n, taking the divergence of Equation (17) and imposing the incompressibility condition at n+1:

(20) $$\nabla \;∙\;u^{n+1}=0$$

results in a pressure‐Poisson Equation:

(21) $$\nabla^{2}\left(p^{n+1}-p^{n}\right)=\frac{\rho }{\Delta t}\nabla ∙\;u^{*}.$$

This equation is numerically solved via preconditioned conjugate gradient solvers which employ deflation in combination with diagonal or linelet preconditioning for acceleration, which implies that the result does not depend on the timestep Δt.

c *Velocity correction* $u^{*}\to u^{n+1}$:

The velocity at the new step is then found from Equation (17):

(22) $$u^{n+1}=u^{*}-\frac{\Delta t}{\rho }\nabla \left(p^{n+1}-p^{n}\right).$$

Equations (18), (21), and (22) are discretized in space using finite elements based on linear tetrahedra and edge‐based data structures to optimize the code performance. Furthermore, to obtain fast steady‐state results, the following techniques have been implemented [34, 35, 36]: (i) local timesteps, (ii) reduced iteration for the pressure, and (iii) substepping for the advection terms. More details about the flow solver can be found in [37, 38].

At the vessel inlet, a velocity profile (parabolic for steady flow, Womersley for pulsatile flow) is mapped to the inlet mesh points. At the outlet(s) traction‐free or 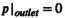 boundary conditions are imposed, while Neumann natural boundary conditions are prescribed at the inlet for the pressure equation (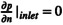). No‐slip boundary conditions are imposed at the vessel walls (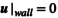). Similarly, using an immersed boundary approach no‐slip boundary conditions are imposed at the intersection between the mesh edges and the device wires.

#### 2.3.4 Transport Solver

The transport equations are numerically solved using a finite volume approach. Integrating the differential equation over the volume of a tetrahedral element yields:

(23) $$\int \frac{\partial C_{i}}{\partial t}\mathrm{dV}+\int \nabla ∙\left(C_{i}u\right)\mathrm{dV}-\int C_{i}\nabla ∙u\;\mathrm{dV}=\int \nabla ∙\left(D_{i}\nabla C_{i}\right)\mathrm{dV}+\int S_{i}\mathrm{dV}$$

where the convective term has been written in conservative form using the fact that:

(24) $$\nabla ∙\left(C_{i}u\right)=u∙\nabla C_{i}+C_{i}\nabla ∙u.$$

Moving the second and third terms on the left to the right‐hand side (RHS) and using the divergence (Gauss') theorem gives:

(25) $$\int \frac{\partial C_{i}}{\partial t}\mathrm{dV}=-∯C_{i}\left(u∙n\right)\mathrm{dA}+∯D_{i}\nabla C_{i}∙n\;\mathrm{dA}+\int C_{i}\nabla ∙u\;\mathrm{dV}+\int S_{i}\mathrm{dV}$$

where n is the unit vector in the direction of the outer normal to the volume surface. The first term on the RHS represents convective flux across the surface of the element, the second term is the diffusive flux across the element surface, the third term is an added source to account for the fact that the velocity field may not be exactly zero, and the last term represents the total production/reaction in the element. Now, assuming a cell‐centered approximation where the concentrations are assumed constant over the element volume yields (for clarity, the species subindex i is converted to a superscript):

(26) $$V_{k}\frac{\partial C^{i}}{\partial t}=-\sum_{j=1}^{N_{\text{face}}}C_{j}^{i}\;\left(u_{j}∙n_{j}\right)A_{j}+\sum_{j=1}^{N_{\text{face}}}D_{j}^{i}\left(\nabla C_{j}^{i}∙n_{j}\right)\;A_{j}+C_{k}^{i}\;Q_{k}V_{k}+S_{k}^{i}\;V_{k}$$

where $V_{k}$ is the volume of element k, j loops over the faces of element k ($N_{\text{face}}=4$ for tetrahedra), $n_{j}$ is the (outer) normal to face j, $A_{j}$ is the area, and $u_{j}$ is the average velocity at the face. The total volume flow ($Q_{k}$) into element k is calculated as:

(27) $$Q_{k}=\int \nabla ∙u\;\mathrm{dV}=∯\left(u∙n\right)\;\mathrm{dA}=\sum_{j=1}^{N_{\text{face}}}\left(u_{j}∙n_{j}\right)\;A_{j}.$$

The diffusivity at the face is taken as the average diffusivity of the two adjacent elements $k_{1}$ and $k_{2}$:

(28) $$D_{j}^{i}=\frac{1}{2}\left(D_{k_{1}}^{i}+D_{k_{2}}^{i}\right)$$

and the diffusive flux at the face is approximated as:

(29) $$\left(\nabla C_{j}^{i}∙n_{j}\right)=\frac{C_{k_{1}}^{i}-C_{k_{2}}^{i}}{\Delta s_{j}}$$

where $\Delta s_{j}$ is the normal distance between the centroids of the two elements $k_{1}$ and $k_{2}$ adjacent to face j. This scheme is equivalent to a second‐order central difference approximation for the diffusive flux.

To obtain a stable numerical scheme, the convective term must be discretized with an upwind strategy [39]. The simplest approach is to take the “upwind” concentration at the face, that is, assuming that the normal vector at face j goes from $k_{1}$ to $k_{2}$ (see Figure S1) the face concentration is taken from the “upstream” element:

(30) $$C_{j}^{i}=\left\{\begin{array}{cc} C_{k_{1}}^{i} & \text{if}\;\left(u_{j}∙n_{j}\right)>0\\ C_{k_{2}}^{i} & \text{if}\;\left(u_{j}∙n_{j}\right)<0 \end{array}\right..$$

This is equivalent to a first‐order upwind method. Now, the scheme can be made second order accurate by using a total variation diminishing (TVD) scheme based on variable extrapolation [39], which is described in detail in Appendix A.

The finite volume Equation (26) is integrated in time using an explicit Runge–Kutta method. The timestep must obey the following stability conditions:

(31) $$\begin{array}{ll} \text{convection}: & \Delta t<h_{k}/\left|u_{k}\right|\\ \text{diffusion}: & \Delta t<h_{k}^{2}/\left(2D_{k}^{i}\right)\\ \text{divergence}: & \Delta t<V_{k}/Q_{k}\\ \text{reactions}: & \Delta t<\tau_{k}^{\mathrm{ij}} \end{array}$$

where $h_{k}$ is the size of the element k (e.g., $h_{k}=\sqrt[{3}]{V_{k}}$) and $\tau_{k}^{\mathrm{ij}}$ represents the characteristic reaction time between species i and j in element k. The timestep is then calculated at each step as:

(32) $$\Delta t=\mathrm{Cou}*\Delta t_{\min}$$

where $\Delta t_{\min}$ is the minimum of the expressions in Equation (31) over all elements and species, and Cou (Courant number) is a factor below one.

Boundary conditions are imposed by prescribing the flux through boundary faces. For faces at the inlet(s), only convective fluxes are computed (diffusive flux is assumed zero) with the “upstream” concentration taken from the prescribed boundary value, which in general could be a function of time given in a table: 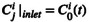. Similarly, for faces at the outlet(s), the convective flux is computed with the concentration of the “upstream” element: 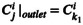. The vessel walls are assumed impermeable, and thus, both the convective and dissuasive fluxes are set to zero. Similarly, no flux boundary conditions are assumed through faces cut by device wires (i.e., immersed boundaries).

Finally, for “bounded” species (e.g., i=Fb), convective and diffusive fluxes are set to zero. However, to allow bounded fibrin to propagate to nearby elements (otherwise it would not be possible to reproduce device occlusions observed experimentally), the concentration of bounded fibrin used in the reaction Equation (15) of element k is computed as the average from element k and the neighboring elements contained within a specified distance roughly equivalent to the length of fibrin fibers (e.g., 40μm):

(33) $$C_{F_{b}}\left(k\right)=\frac{1}{N_{\text{neig}}\left(k\right)}\sum_{j=1}^{N_{\text{neig}}\left(k\right)}C_{\mathrm{Fb}}\left(j\right).$$

#### 2.3.5 Coupling

The solution of the coupled system proceeds in a staggered manner by alternatively running the flow and transport solvers. To simplify the simulation, a quasi‐steady strategy is adopted where the fluid flow Equations (18) are advanced until a steady state is reached under prescribed mean flow conditions at the vessel inlet.

The criterion for convergence to a steady state can be written as:

(34) $$\mathrm{\delta u}=\left|u^{n+1}-u^{n}\right|=\Delta t*|R_{1}+R_{2}|<\epsilon_{1}$$

where $\epsilon_{1}$ is a tolerance threshold, and the residual $R=R_{1}+R_{2}$ has been split into two contributions: (1) the residual of the Navier–Stokes' equations without the porosity term:

(35) $$R_{1}=-u^{n}∙\nabla u^{n}-\frac{1}{\rho }\nabla p^{n+1}+\frac{\mu }{\rho }\;\nabla^{2}u^{n}$$

and (2) the contribution from the porosity force:

(36) $$R_{2}=-\frac{\pi }{\rho }\phi \;u^{n+1}.$$

Next, using the resulting velocity field u, the shear stress τ is calculated using Equation (5), and the transport Equations (26) are advanced assuming the flow solution does not change (i.e., u and τ fields are kept constant). As the concentration fields change, the porosity force of Equation (7) is updated and the calculation stops when the change in this force is considered large enough to require a new flow solution. Denoting m the current timestep of the transport calculation and m=0 is the initial step, since the velocity, shear stress, and pressure fields are assumed fixed, $R_{1}$ is also fixed, and the change in the fluid residual can be estimated as:

(37) $$\mathrm{\delta R}\approx \Delta t*\delta R_{2}=\Delta t*\mathrm{\delta F}$$

where δF is the change in the porosity force during the transport calculation:

(38) $$\mathrm{\delta F}=\left|F\left(C_{F_{b}}^{m}\right)-F\left(C_{F_{b}}^{0}\right)\right|.$$

If the residual of the fluid equation is only allowed to increase up to a tolerance of $\epsilon_{2}>\epsilon_{1}$, then a new flow solution is required when

(39) $$\mathrm{\delta R}=\frac{\epsilon_{2}}{\Delta t}.$$

In this manner, the flow solution is always converged within a tolerance $\epsilon_{2}$, but at every iteration, the flow solver is required to lower the residual down to below $\epsilon_{1}$. In the simulations presented below, the following thresholds were used: $\epsilon_{2}=10^{-3}$ and $\epsilon_{1}=10^{-4}$.

If desired, the quasi‐steady assumption can be relaxed and flow pulsatility is included in the model by solving the unsteady flow equations for one cardiac cycle and storing the volumetric flow field at a number of time points during the cycle (say 100 instants). Then, during the transport calculation, the flow velocity is interpolated in time from the stored flow fields assuming periodicity over the cardiac cycle. The corresponding shear stress field is then computed and the transport solution is advanced as explained above.

#### 2.3.6 Implementation

In the current implementation, two independent codes, the fluid and transport solvers, are alternatively called from a third program that drives the coupled simulation. At each step of the coupled simulation, the fluid solver reads the element porosities from a binary file, and at the end of its calculation, saves the velocity field to another binary file. The transport solver then reads this velocity field, advances the concentration field in time and when the stopping criteria is met, it saves the concentrations and the porosities to binary files. The process is repeated until a desired number of coupled steps or a global time (the sum of all the transport simulation times) reaches a desired value.

To speed up the simulations, all data necessary to restart the fluid and transport calculations (including meshes, fields, derived data structures such as element faces) are saved to binary files which are read when the codes are reinitialized. Both the fluid and transport solvers have been parallelized for shared memory architectures using OpenMP. In the transport solver, parallelization was implemented by storing fluxes at the element faces in an array and computing these fluxes in parallel since they are independent from one another, and then the element RHS is calculated in parallel by gathering the fluxes from the faces of each element.

Values of model parameters deduced from previous studies [20, 40, 41, 42] and tuned (by trial and error) to reproduce the fibrin accumulation patterns observed in experiments are listed in Tables S1–S4. of Appendix B.

## 3 Results

### 3.1 Straight Tube

The methodology was first tested on a simple model of the flow in a straight circular cylinder with a device mesh placed perpendicularly to the flow at the center of the tube. The tube had a diameter of 300μm and a length of 3mm. At the inlet, a parabolic velocity profile was prescribed with a mean velocity of 1.5cm/s. At the outlet, a traction‐free (zero‐pressure) boundary condition was applied. Physiologic concentrations of prothrombin ($C_{\mathrm{PT}}=1.4\;\mathrm{\mu M}$), antithrombin ($C_{\mathrm{AT}}=2.41\;\mathrm{\mu M}$), and fibrinogen ($C_{F_{g}}=7.0\;\mathrm{\mu M}$) were prescribed at the inlet [20, 42], while the concentrations of other species (thrombin and fibrin) were kept at zero. All concentrations were initialized to zero.

**FIGURE 3:**
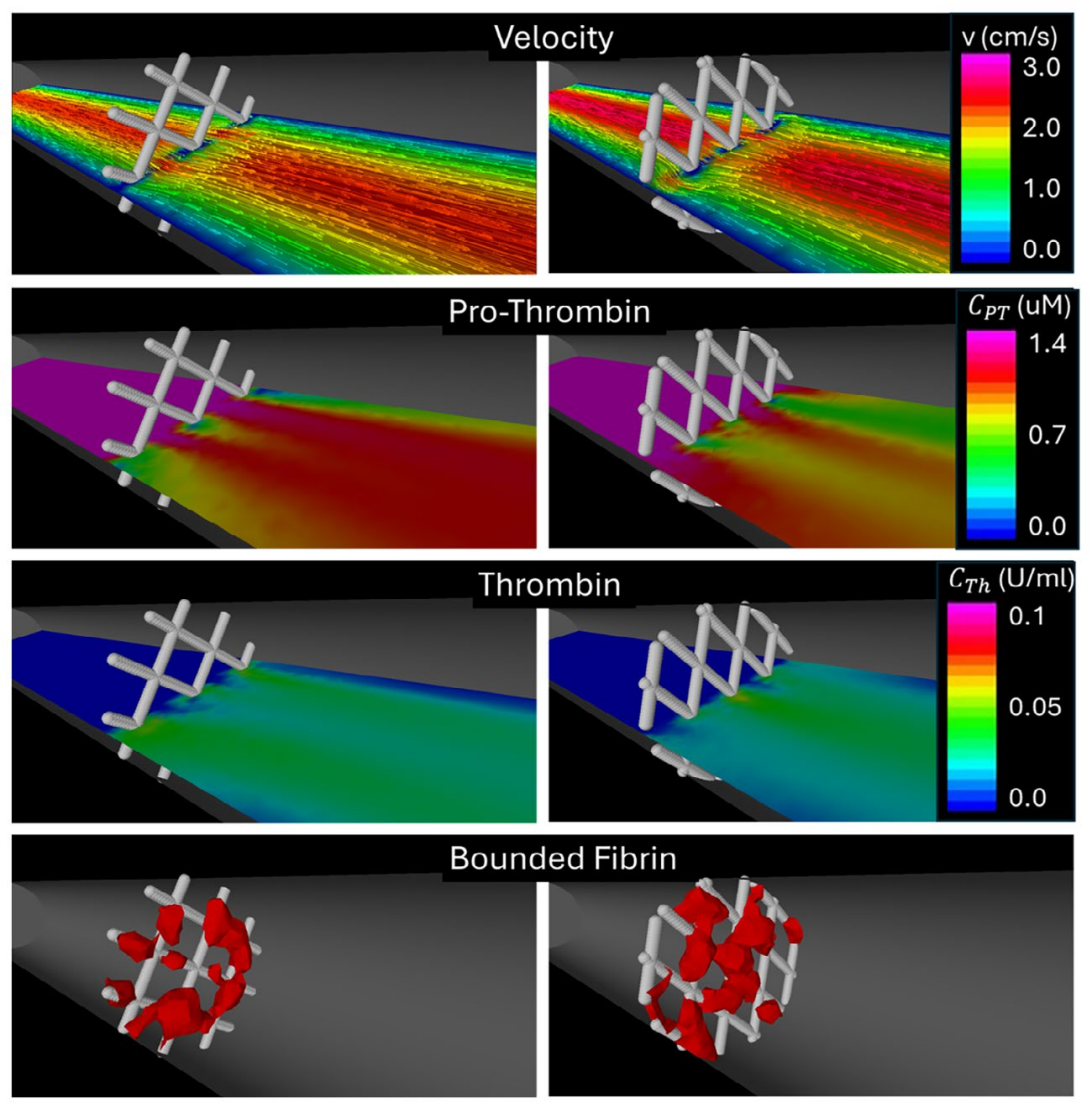
Fibrin accumulation on device cells with different pore sizes placed perpendicularly to the flow in a straight tube. From top to bottom: velocities on a horizontal cut plane, concentration of prothrombin on the cut plane, concentration of thrombin on the cut plane, and isosurfaces of bounded fibrin concentration. Results are presented at a final time of approximately 20 min.

Results were obtained for two device meshes with different pore sizes corresponding to cells with an internal angle of $90^{o}$ and $150^{o}$, respectively. The wire thickness was 20μm. The results presented in Figure 3 show the flow alteration produced by the device wires, the consumption of prothrombin and production of thrombin, and the accumulation of (bounded) fibrin on the device wires. The results suggest a larger accumulation of fibrin in devices with smaller pore sizes (larger braid angles).

### 3.2 3D‐Printed Sidewall Aneurysm Model

A flow diverter was deployed in an experimental model of a sidewall aneurysm of 8mm diameter created by 3D printing. The parent vessel had an internal diameter of 4mm and a radius of curvature of 4cm. The flow diverter had a braided design with 48 wires with a thickness of 30μm and a braid angle of $90^{o}$. The model was connected to a flow loop with a peristaltic pump circulating human plasma from a reservoir at a flow rate of 4cc/s. The fibrin attached to the device wires covering the neck of the aneurysm was visualized and quantified at different times.

**FIGURE 4:**
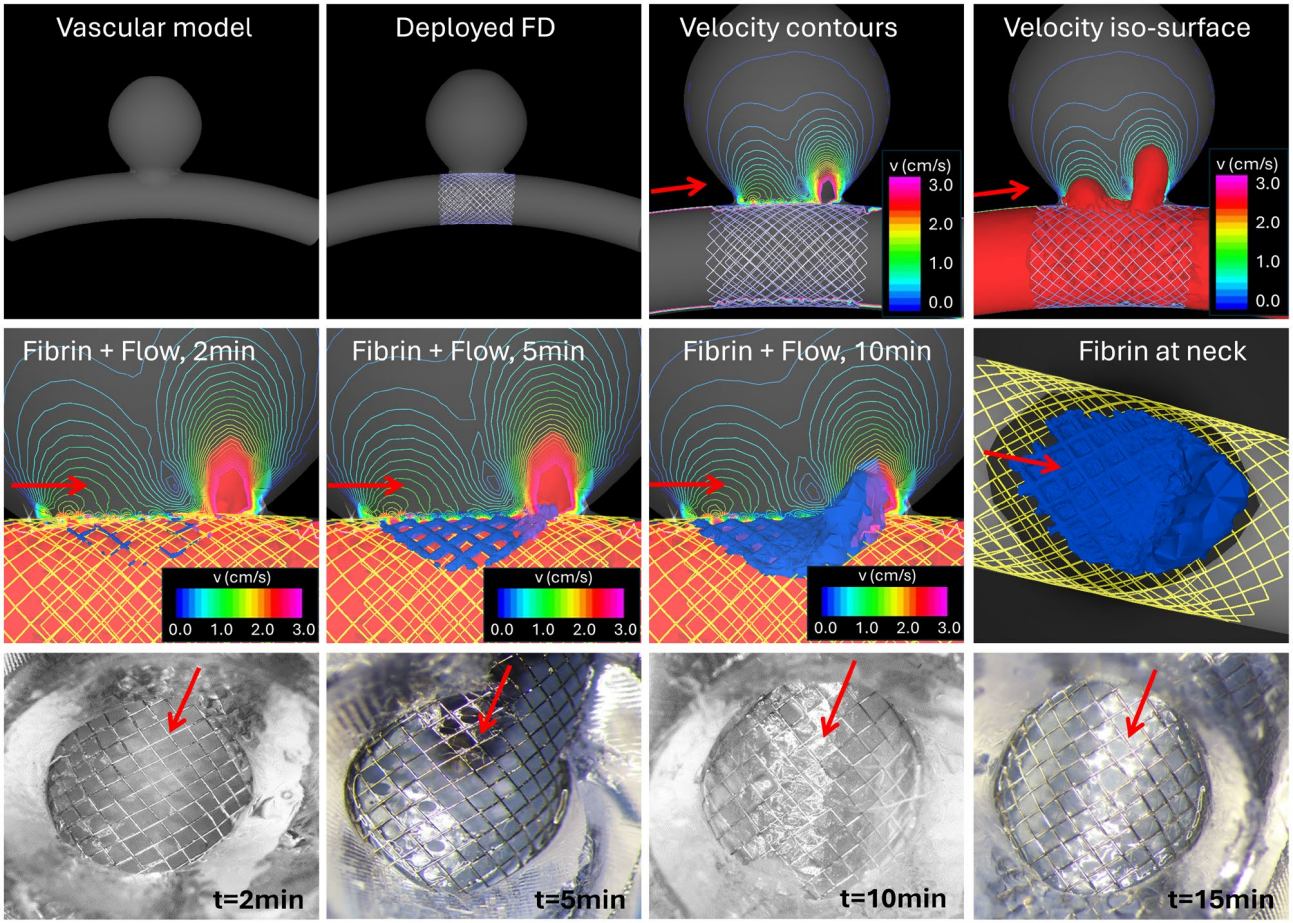
Fibrin accumulation on FD device in the idealized 3D‐printed model. Top row, left to right: vascular model, implanted FD device, velocity field on cut‐plane, velocity isosurface (*v* = 2 cm/s). Middle row, left to right: accumulation of fibrin (blue isosurface) and corresponding flow modification at three different time points, and final fibrin accumulation at aneurysm neck. Bottom row, left to right: fibrin accumulated at the neck of experimental models at four different time points. Red arrows indicate flow direction.

A corresponding computational model was created from the STL file used to 3D print the experimental model. Steady inflow conditions were prescribed by mapping a parabolic velocity profile with a steady flow rate of 4 cc/s. Traction‐free boundary conditions were applied at the outlet. A corresponding FD device model was created and virtually deployed within the computational model. The top row of Figure 4 presents (from left to right), the computational model showing the curvature of the parent artery, the deployed FD device covering the aneurysm neck, and visualizations of the postimplantation flow velocity using contours on a cut‐plane and a velocity isosurface. These visualizations show the inflow stream at the distal part of the neck (flow is from left to right) and the outflow toward the proximal part of the neck.

The middle row of Figure 4 shows the accumulation of (bounded) fibrin at the device wires (rendered as an isosurface of $F_{b}$ concentration) at three different times along with the reduction of the flow velocity into the aneurysm, and the final accumulated fibrin at the aneurysm neck. Experimental results obtained at four different times are presented in the bottom row of Figure 4 (flow direction is indicated by red arrows). It can be seen that the computational and experimental results qualitatively agree. Specifically, fibrin starts to accumulate at the distal part of the neck coinciding with the aneurysm inflow, and then toward the proximal part of the neck (outflow). As fibrin accumulates, the flow into the aneurysm is further disrupted and reduced.

Note that in these 3D‐printed experimental models, visualization of fibrin accumulation at the device wires was performed by terminating different experiments at different times and sectioning the models to show the device wires at the aneurysm orifice. These are the images provided at the bottom row of Figure 4, and as such, they do not correspond to a single experiment but to different models (with the same 3D printed geometry) run for different total times. Thus, the presence of device cells with little or no fibrin at 15 min does not necessarily imply fibrin detachment (which is not currently included in the computational model), and it simply means that in some of these experiments, these cells were not fully covered.

### 3.3 Glass Model of Sidewall Aneurysm

In the previous experiments described above that used blood plasma in a 3D‐printed model, it was difficult to observe the fibrin accumulation due to the turbidity of the mixture. Therefore, new experiments were carried out using a clear fibrinogen concentrate (Fibryga, Octapharma USA, NJ, USA) as the experimental fluid in a transparent glass model [42, 43]. Furthermore, the use of this experimental fluid allowed the study of the effects of fibrinogen alone. The fibrinogen concentrate was diluted to physiologic values ($C_{F_{g}}=330\;\mathrm{mg}/\mathrm{dl}$) and circulated at 4cc/sec. Similar to the previous experimental model, an 8−mm spherical aneurysm was created in a 4−mm vessel with a radius of curvature of 5cm, but in this glass model, it resulted in a wider neck. A flow diverter of 4.75mm diameter, 20mm length and composed48 wires of 32μm thickness was deployed across the aneurysm neck. Qualitative flow visualization was performed by injecting a purple dye and observing it circulate around the aneurysm.

**FIGURE 5:**
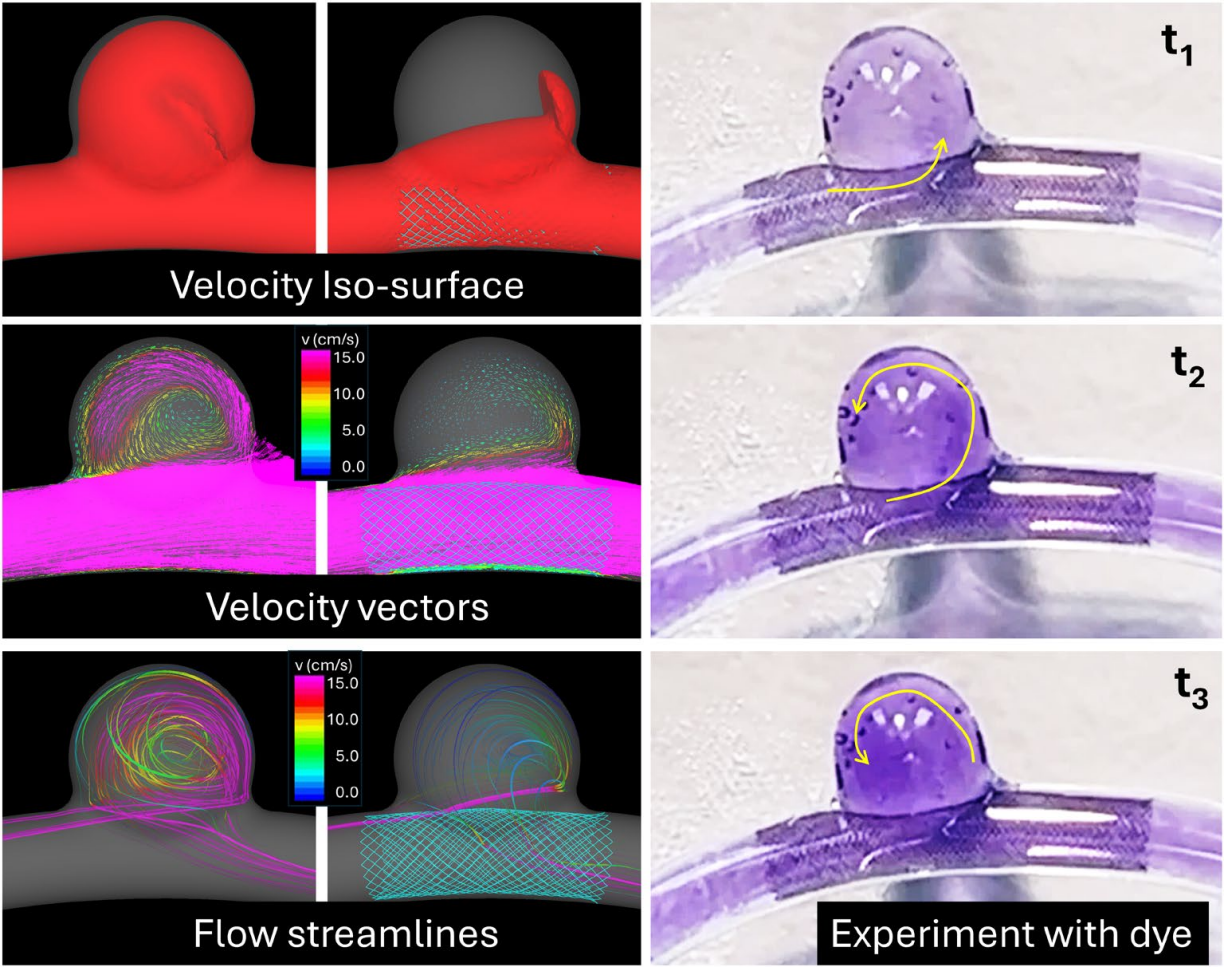
Flow visualization before (left column) and after FD device implantation in the computational model (center column) of sidewall aneurysm. Right column: flow visualization in the experimental model at three instants of time ($t_{1}<t_{2}<t_{3}$) using a purple dye (yellow arrows indicate dye movement inside the aneurysm).

A corresponding computational model was created from a 3D scan of the glass model. As in the previous model, steady inflow conditions were prescribed by mapping a parabolic velocity profile with a steady flow rate of 4 cc/s and traction‐free boundary conditions were applied at the outlet. Flow visualizations are presented in Figure 5. The left column shows, from top to bottom, isovelocity surface (*v* = 5 cm/s), velocity vectors on the center cut‐plane, and flow streamlines, obtained with the computational model before deploying the FD device. The center column shows corresponding visualizations after deployment of the FD device. It can be seen that the FD device reduced the velocity into the aneurysm, but the flow pattern still had the inflow at the distal neck and the outflow toward the proximal part of the neck. This flow pattern is consistent with the experimental flow visualizations presented in the right column of Figure 5 which show the contrast entering the aneurysm at the distal neck ($t_{1}$) and then recirculating in a counterclockwise direction ($t_{2},t_{3}$) as indicated by the yellow arrows that trace the darker purple regions of higher dye concentration.

**FIGURE 6:**
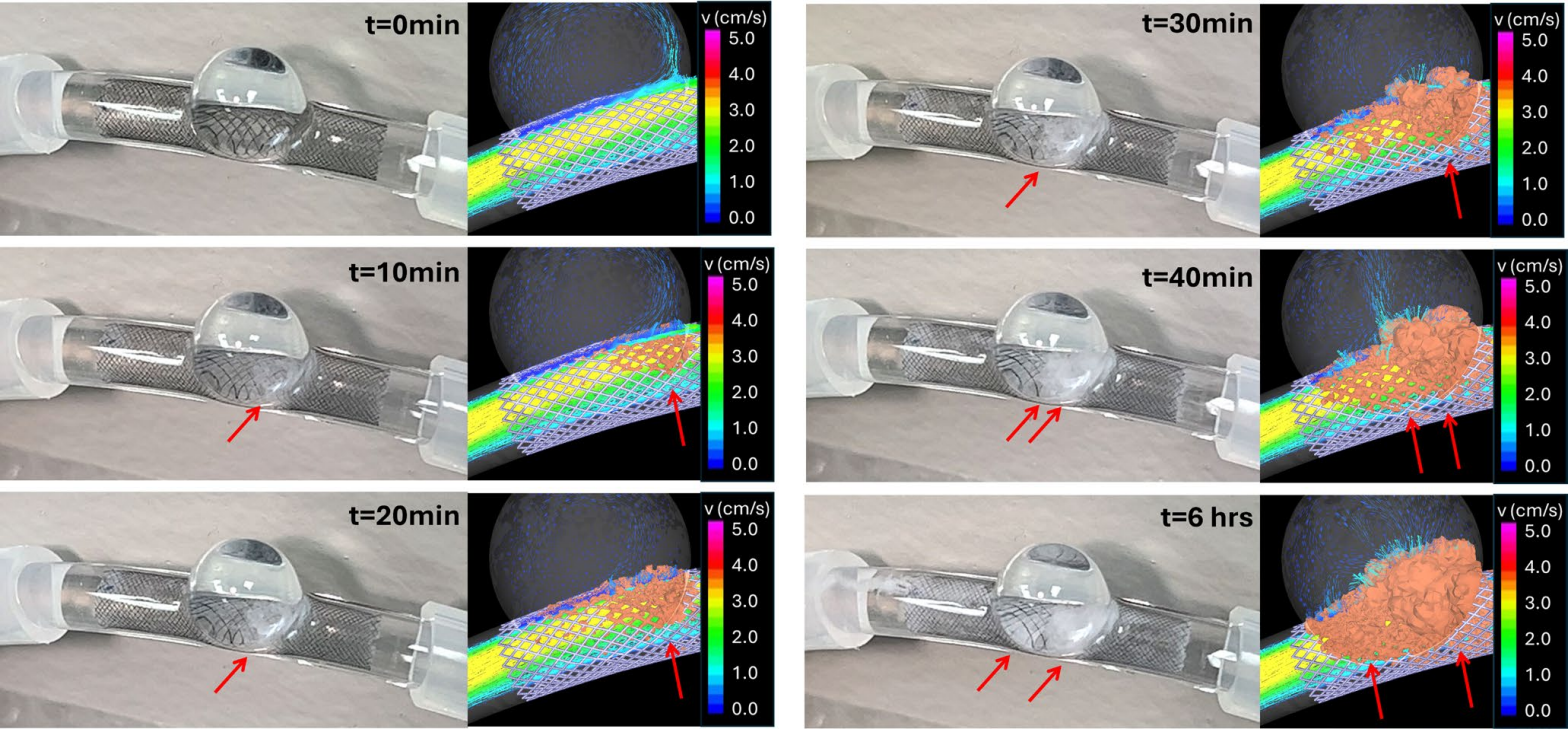
Visualization of progressive fibrin accumulation in glass model of stented sidewall aneurysm and a corresponding computational model showing isosurfaces of fibrin attached to the wires (orange) and corresponding flow modification. Red arrows point to white opaque regions corresponding to fibrin accumulation in the in vitro models and corresponding bounded fibrin isosurfaces in the computational model.

The fibrin accumulation experiments performed with this system revealed two interesting facts: (a) there was no fibrin production/accumulation in the absence of flow, and (b) fibrin was produced and accumulated even in the absence of thrombin. Thus, to mimic the experimental setup, the computational model in this case only considered fibrin production stimulated from flow shear stress, and adhesion to the device wires and/or already bounded fibrin. Experimental and computational results of fibrin accumulation at different times are shown in Figure 6. The computational results are consistent with the experimental observations. Specifically, fibrin starts accumulating in the inflow zone of the aneurysm orifice, toward the distal part of the neck, and as time progresses it continues to accumulate in this region but also toward the proximal part of the neck. Again, as fibrin accumulates, it can be seen how the inflow stream is further disrupted and its velocity reduced.

## 4 Discussion

Understanding the relative importance and precedence of processes such as thrombus formation within the aneurysm cavity and fibrin accumulation at the FD cells across the neck, and the associated disruption of the aneurysm inflow and intra‐aneurysmal flow slow down are important for improving FD devices and assessing outcomes of FD procedures. Computational and experimental models provide useful insights into these processes. Previous work has mainly focused on intra‐aneurysmal thrombus formation and has described the role of platelets and fibrin in the formation of intra‐aneurysmal clots [20, 41]. The current study focused on modeling the production of fibrin and its adhesion to FD wires and subsequent accumulation across the aneurysm neck and associated inflow alteration.

This article described in detail a computational model that was developed to study these processes, with guidance from in vitro experiments and observations. In particular, in vitro experiments conducted using a fibrinogen concentrate [42, 43] revealed that fibrin does not accumulate without flow but is produced and accumulates with flow, which suggested a production term dependent on local flow shear stress. Similarly to previous approaches [20, 41], the coagulation cascade was simplified, and different substances were modeled with a continuous approach. To isolate the effects of fibrin accumulation, no platelets and blood cells were included at this point. In contrast with these previous studies, the current model included thrombin production from prothrombin stimulated by the presence of thrombogenic metallic wires (part of the intrinsic coagulation cascade), production of fibrin from fibrinogen stimulated by flow shear stress (revealed by experiments), and adhesion of fibrin to the FD wires.

The computational approach was demonstrated in idealized vascular geometries and compared with idealized in vitro aneurysm models treated with flow diverters. The results indicate that overall, the computational and experimental models are in good agreement. First, the flow patterns obtained before and after FD device deployment were consistent with the corresponding flow visualization using dyes in the in vitro models. Second, the progressive production and accumulation pattern of fibrin were consistent between the computational model and experiments conducted with blood plasma. Third, fibrin production based on flow shear stress also yielded results consistent with experimental observations of fibrin accumulation using fibrinogen concentrate alone.

The current study has several limitations. The coagulation cascade was considerably simplified, and substances (including fibrin) and their interactions were approximated with a continuous approach. Although this is a limitation, it enables the control and isolation of different effects such as fibrin production from flow shear stress. Of course, the model can be made more sophisticated by adding more species and reactions to account for other interactions (for instance, the effects of drugs such as heparin). The model includes several parameters, mainly rate constants, that are difficult to determine individually. The current values were initially taken from previous reports and subsequently tuned by trial and error to reproduce the fibrin accumulation patterns observed in experiments. Further fine‐tuning of these parameters would require more and more controlled experiments. The current coupled approach is not exactly time accurate since, for example, the flows are converged to steady state within each coupled iteration. This limitation can be somewhat relaxed, for example, by computing blood flows over one cardiac cycle and assuming periodicity, or the flow and transport solvers could be more tightly coupled to advance the coupled solution in time more accurately. However, this would result in even more computationally expensive simulations. Although the codes have been parallelized and use specialized data structures to optimize the performance, fibrin deposition calculations are still quite computationally expensive requiring long simulation times. For example, one of the most expensive simulations consisting of 100 coupled steps with 200 flow steps and 3000 transport steps each and a mesh size of approximately 5 million elements required approximately 60 h running on 18 cores. Despite these limitations, the current study showed that the computational model was able to reproduce the fibrin dynamic behavior observed in experimental in vitro models and therefore can be used to better understand the effects leading to fibrin accumulation and the resulting aneurysm inflow reduction.

## 5 Conclusions

A coupled computational model of fibrin accumulation and flow modification after flow diversion treatment of cerebral aneurysms has been developed under the guidance of in vitro experiments and observations. The model was able to reproduce the salient features of fibrin accumulation after deployment of FD devices in idealized in vitro models of cerebral aneurysms.

## Ethics Statement

The authors have nothing to report.

## Conflicts of Interest

The authors declare no conflictS of interest.

## Supporting information


Data S1–S4.

## Data Availability

The data that support the findings of this study are available from the corresponding author upon reasonable request.
