# Supplementary material for: Modeling Fibrin Accumulation on Flow‐Diverting Devices for Intracranial Aneurysms
Source: Int J Numer Method Biomed Eng. 2024 Nov 5;40(12):e3883. doi: 10.1002/cnm.3883 (PMC11618230; doi:10.1002/cnm.3883)
Supplement: Supplementary file 1 — Data S1–S4. [file CNM-40-e3883-s001.docx]

**SUPPLEMENTARY MATERIAL**

**APPENDIX A: TVD Upwind Finite Volumes Scheme**

Consider the triangular face $j$ with normal $\boldsymbol{n}_{j}$ between two tetrahedral elements $k_{1}$ and $k_{2}$, as shown in Supplementary Figure 1.


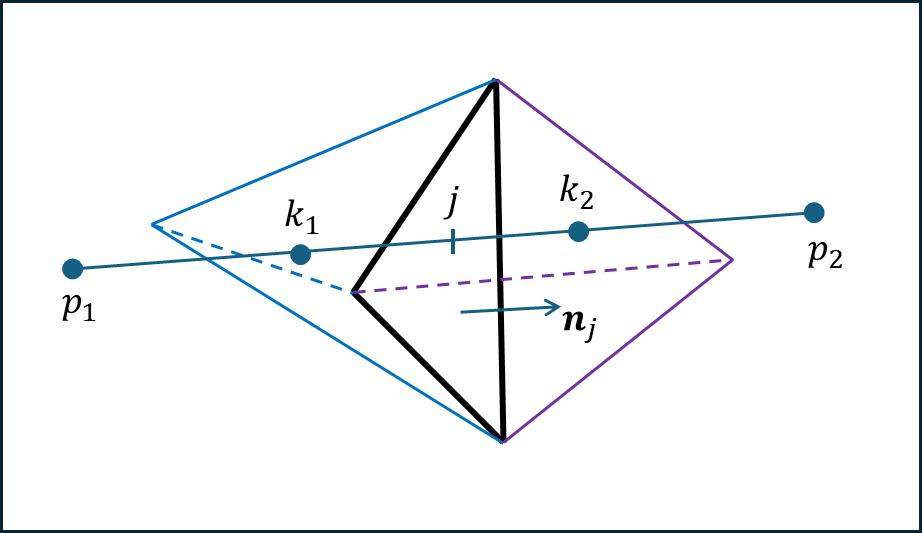


**Supplementary Figure 1:** Flux through triangular face ($j$) with two adjacent elements ($k_{1}$ and $k_{2}$).

The convective flux across this face is, as shown in equation (26):

| $Flux_{j}=C_{j}^{i} \boldsymbol{u}_{j}\cdot\boldsymbol{n}_{j}$ | (40) |
| --- | --- |

where the concentration at the face is taken from the upstream element for a first order upwind scheme, as indicated in equation (30). In order to make the scheme second order accurate, the concentration at the face is extrapolated from the upstream direction to the face. Since in the cell-centered finite volume approach the concentration is constant at the element, the concentration at an upstream location can be computed from a Taylor series approximation. Assuming $\boldsymbol{u}_{j}\cdot\boldsymbol{n}_{j}>0$, i.e. upstream to the left, downstream to the right of the figure, the concentration at an upstream location $p_{1}$ is taken as:

| $C_{p_{1}}^{i}=C_{k_{1}}^{i}-\nabla C_{k_{1}}^{i}\cdot\boldsymbol{n}_{j} ds$ | (41) |
| --- | --- |

where $ds$ is the normal distance between the centroids of the elements, and the concentration at the face is calculated as:

| $C_{j}^{i}=C_{k_{1}}^{i}+\frac{1}{2} \psi\left( r_{1}^{i} \right) \left( C_{k_{1}}^{i}-C_{p_{1}}^{i} \right)$ | (42) |
| --- | --- |

where $\psi$ is a limitor function to make the scheme total-variation-diminishing (TVD) by avoiding over and under shoots, with the ratio $r_{1}^{i}$ given by:

| $r_{1}^{i}=\left\{ \begin{matrix} \left\vert C_{k_{2}}^{i}-C_{k_{1}}^{i} \right\vert/\left\vert C_{k_{1}}^{i}-C_{p_{1}}^{i} \right\vert& if \left\vert C_{k_{1}}^{i}-C_{p_{1}}^{i} \right\vert>0 \\ 0 & otherwise \end{matrix} \right.$ | (43) |
| --- | --- |

Similarly, if $\boldsymbol{u}_{j}\cdot\boldsymbol{n}_{j}<0$ (velocity from right to left), the concentration at an upstream location $p_{2}$ is:

| $C_{p_{2}}^{i}=C_{k_{2}}^{i}+\nabla C_{k_{2}}^{i}\cdot\boldsymbol{n}_{j} ds$ | (44) |
| --- | --- |

and with the ratio $r_{2}^{i}$ defined as:

| $r_{2}^{i}=\left\{ \begin{matrix} \left\vert C_{k_{2}}^{i}-C_{k_{1}}^{i} \right\vert/\left\vert C_{p_{2}}^{i}-C_{k_{2}}^{i} \right\vert& if \left\vert C_{p_{2}}^{i}-C_{k_{2}}^{i} \right\vert>0 \\ 0 & otherwise \end{matrix} \right.$ | (45) |
| --- | --- |

the face concentration becomes:

| $C_{j}^{i}=C_{k_{2}}^{i}-\frac{1}{2} \psi\left( r_{2}^{i} \right) \left( C_{p_{2}}^{i}-C_{k_{2}}^{i} \right)$ | (46) |
| --- | --- |

Different forms of the limitor function have been defined, for example:

| $\psi\left( r \right)=\max\left( 0,\min\left( \beta r,1 \right),\min\left( r,\beta\right) \right)$ | (47) |
| --- | --- |

where the parameter $\beta=2$ for the Superbee scheme, $\beta=1$ for the minmod scheme, and $1<\beta<2$ in Sweby’s scheme. Best results were obtained with the Superbee scheme. Also note that the first order upwind scheme is recovered when $\psi\left( r \right)=1$.

The gradient of the concentration at a given element $k$, needed in equations (41) and (44), is found by the application of Gauss theorem to a vector field $\boldsymbol{w}$:

| $\int\nabla\cdot\boldsymbol{w} dV=∯ (\boldsymbol{w}\cdot\boldsymbol{n}) dA$ | (48) |
| --- | --- |

Taking this vector field as the concentration times the unit vector along the coordinate direction $l$, yields the partial derivative along $x_{l}$:

| $\boldsymbol{w}=C^{i} {\hat{\boldsymbol{x}}}_{l} \Rightarrow\nabla\cdot\boldsymbol{w}=\frac{\partial C^{i}}{\partial x_{l}}$ | (49) |
| --- | --- |

Assuming this derivative to be constant in the element yields:

| $V_{k} \left( \frac{\partial C^{i}}{\partial x_{i}} \right)_{k}=∯ C^{i} {\hat{\boldsymbol{x}}}_{l}\cdot\boldsymbol{n} dA=∯ C^{i} dA_{l}$ | (50) |
| --- | --- |

and it is then computed by summing over the element faces

| $\left( \frac{\partial C^{i}}{\partial x_{i}} \right)_{k}=\frac{1}{V_{k}} \sum_{j=1}^{N_{face}} C_{j}^{i} \left( dA_{j} \right)_{l}$ | (51) |
| --- | --- |

where $\left( dA_{j} \right)_{l}$ is the component along the $l$ coordinate direction of the area element of face $j$, and the concentration at the face is here taken as:

| $C_{j}^{i}=\frac{1}{2}\left( C_{k_{1}}^{i}+C_{k_{2}}^{i} \right)$ | (52) |
| --- | --- |

**APENDIX B: Model Parameters**

The parameters used in the fluid calculations are provided in Supplementary Table 1.

| Parameter | Symbol | Value |
| --- | --- | --- |
| Density | $\rho$ | $1.0 g/cm^{3}$ |
| Viscosity | $\mu$ | $0.012 g/cm.s$ |
| Porosity | $\pi$ | $5\times{10}^{3} g/s$ |

**Supplementary Table 1**: Fluid parameters.

The values of the initial and/or inlet concentration and diffusivities of the different species are given in Supplementary Table 2.

| Species | Symbol | Initial / Inlet  Concentration | Diffusivity |
| --- | --- | --- | --- |
| Anti-thrombin | $AT$ | $2.41 \mu M$ | $5.57\times{10}^{-7} cm^{2}/s$ |
| Pro-thrombin | $PT$ | $1.40 \mu M$ | $5.21\times{10}^{-7} cm^{2}/s$ |
| Thrombin | $T_{h}$ | $0.1 U/ml$ | $6.47\times{10}^{-7} cm^{2}/s$ |
| Fibrinogen | $F_{g}$ | $7.0 \mu M$ | $3.10\times{10}^{-7} cm^{2}/s$ |
| Fibrin (free) | $F_{n}$ | $0.0$ | $2.47\times{10}^{-7} cm^{2}/s$ |
| Bounded fibrin | $F_{b}$ | $0.0$ | $0.0$ |

**Supplementary Table 2**: Species concentrations and diffusivities.

The thresholds and exponents of the Hill functions used in the source terms and the porous force are given in Supplementary Table 3.

| Function | Threshold | Value | Exponent |
| --- | --- | --- | --- |
| Wire distance | $r_{0}$ | $20 \mu m$ | 4 |
| Shear stress production | $\tau_{0}$ | $200 dyn/cm^{2}$ | 10 |
| Bounded fibrin porosity | $C_{F_{b0}}$ | $0.1 \mu M$ | 4 |
| $F_{g}$ / thrombin stimulation | $K_{m}$ | $3.16 \mu M$ | - |

**Supplementary Table 3**: Threshold parameters and Hill function exponents.

The rate constants for the different interactions included in the model are given in Supplementary Table 4. Since the flow and transport equations are not solved in a time accurate fashion, i.e. flow is converged to steady state at each coupled step and maintained constant during the transport calculation, the rate constants are given in arbitrary time units ($t$), where the final simulation time of $t=0.4$ is roughly equivalent to the $6 hrs$ of the in vitro experiments presented in Figure 6.

| Rate Constant | Symbol | Value |
| --- | --- | --- |
| Thrombin production by wires | $K_{wt}$ | $0.017 t^{-1}$ |
| Anti-thrombin inhibition | $K_{at}$ | $0.007 \mu M^{-1} t^{-1}$ |
| Thrombin stimulation | $K_{th}$ | $5.9 t^{-1}$ |
| Fibrin production by shear | $K_{ss}$ | $0.02 t^{-1}$ |
| Fibrin adhesion to wires | $K_{wa}$ | $0.5 t^{-1}$ |
| Fibrin adhesion to fibrin | $K_{b}$ | $0.5 \mu M^{-1} t^{-1}$ |

**Supplementary Table 4**: Interaction rate constants.
